# Design and Development of High-Performance Bio-Based Thermoplastic Polyurethane (TPU) Nanocomposites Enabled by Silane-Modified Nanocellulose

**DOI:** 10.3390/polym18131665

**Published:** 2026-07-05

**Authors:** Nello Russo, Federica Recupido, Loredana Tammaro, Maria Oliviero, Barbara Liguori, Roberta Marzella, Letizia Verdolotti, Giuseppe Cesare Lama

**Affiliations:** 1 Institute of Polymers, Composites and Biomaterials, National Research Council of Italy (IPCB-CNR), Piazzale E. Fermi, 1, 80055 Naples, Italy; nellorusso@cnr.it (N.R.); federica.recupido@cnr.it (F.R.); roberta.marzella@cnr.it (R.M.); giuseppecesare.lama@cnr.it (G.C.L.); 2 Department of Chemical, Materials and Industrial Production Engineering (DiCMaPI), University of Naples Federico II, Piazzale V. Tecchio 80, 80125 Naples, Italy; bliguori@unina.it; 3 Laboratory Smart Components and Systems for Sustainable Manufacturing (SSPT-TIMAS-CMS), Italian National Agency for New Technologies, Energy and Sustainable Economic Development (ENEA), Piazzale E. Fermi 1, 80055 Naples, Italy; loredana.tammaro@enea.it

**Keywords:** thermoplastic polyurethane, cellulose nanocrystals, nanocomposites, food packaging, functional materials, sustainability

## Abstract

The food packaging sector widely relies on polymeric materials, and as sustainability concerns grow, commodity polymers need to be replaced with innovative and more sustainable materials. Thermoplastic polyurethane (TPU) is a versatile elastomeric polymer characterized by flexibility, strength, chemical and abrasion resistance, and biocompatibility. However, it presents some limitations, notably in terms of functional properties (i.e., barrier properties). The use of nano-sized renewable fillers, such as cellulose nanocrystals (CNCs), may improve these properties, extending the applicability range of TPU. In this work, bio-based TPU nanocomposites were obtained by adding commercial silane-modified cellulose nanocrystals (Si−O−CNC) at different contents (1–5 wt.%). The nanocomposites were produced via melt mixing followed by compression molding and were characterized in terms of chemical (FTIR), morphological, thermal, mechanical, rheological, wettability, and barrier properties (i.e., water vapor permeability, WVP and oxygen transmission rate, OTR). The presence of Si−O−CNC promoted hydrogen bonding interactions with the TPU matrix, affecting the microphase separation and organization of the hard segments. These microstructural changes improved thermal stability, reduced WVP and OTR, and increased tensile properties at lower nanofiller contents (1–3 wt.%). At higher contents, partial nanofiller aggregation was observed, leading to a reduction in mechanical performance. Overall, these results suggest that TPU/Si−O−CNC nanocomposites have promising potential as sustainable food packaging materials.

## 1. Introduction

Thermoplastic polyurethane (TPU) represents a wide class of elastomeric polymers with applications in various sectors, such as automotive, footwear, biomedical, food packaging, textiles, and sensors [1,2,3]. It is synthesized through the polyaddition of polymeric diols (polyester or polyether) and di-isocyanates (either aliphatic or aromatic) with the aid of chain extenders [4,5,6]. TPU consists of alternating hard segments (HSs), derived from di-isocyanates and short diols, and soft segments (SSs), derived from long-chain diols. HSs provide semi-crystallinity and act as physical crosslinks, while SSs form the amorphous phase, imparting elasticity. Due to the thermodynamic incompatibility between these segments, TPU exhibits a biphasic microstructure [1,4,6,7]. Typically, these precursors are derived from petroleum-based feedstock. However, increasing environmental concerns and the need for sustainable materials have prompted extensive research into renewable alternatives. In this regard, bio-based precursors, mainly polyols obtained from vegetable oils (e.g., castor, olive, soybean, and palm oils [8,9]) as well as from different types of biomass [10,11], or bio-derived di-isocyanates [12,13], have been widely exploited in the literature. Generally, the accurate selection of such precursors allows researchers to attain specific properties in the resulting TPU, such as high tensile strength and flexibility, enhanced abrasion and tear resistance [14,15,16], good solvent resistance, transparency, improved thermal stability [17], and biocompatibility [18]. Nevertheless, TPU usually exhibits limitations such as poor barrier properties, low flame retardancy, and limited antimicrobial properties [19,20,21]. To enhance material performance, various fillers can be incorporated into TPU matrices, including metals (e.g., Ag, Cu nanoparticles, TiO_2_, ZnO [22]), carbonaceous structures (e.g., reduced graphene oxide, carbon nanotubes [23,24]), inorganic materials (e.g., zeolites, clays and diatomite [25,26,27,28]), and organic renewable fillers (e.g., cellulose, starch, and chitosan [29,30]).

Recently, renewable nano-sized fillers such as cellulose nanocrystals (CNCs) have attracted significant attention for their potential use in polymer composites, including TPU-based nanocomposites, due to their high aspect ratio (in the range of 5–50), high strength, biocompatibility, and low toxicity [31,32,33]. They can be extracted from a variety of renewable sources, such as biomass (i.e., bacteria and algae), plants, or animals, exhibiting a rod-like morphology with an average diameter in the range of 3–50 nm [34,35]. In polymer composites, CNCs are mainly employed as mechanical reinforcement [34,36] and to improve barrier properties and thermal insulation [35], whose effectiveness depends on filler-filler and filler-matrix interactions [32]. However, due to the large number of surface hydroxyl groups, CNCs are inherently hydrophilic, which limits their compatibility with hydrophobic polymer matrices and, consequently, negatively affects the material performance. To enhance dispersion and interfacial interactions, surface chemical modifications, such as esterification, silanization, oxidation, etherification, or polymer grafting, are commonly conducted [33,35,36,37,38,39]. Among the surface modification routes, silanization is an inexpensive, easily scalable, and environmentally friendly process, occurring in aqueous media. It involves the chemical grafting of silane coupling agents onto the CNC surface, where they form stable covalent siloxane (Si–O–Si) bonds. This modification introduces hydrophobic moieties or other specific functional groups, thereby enhancing the interfacial adhesion between CNCs and the polymer matrix, while also improving CNC dispersion within polymeric matrices, including polyurethanes [36,37,40]. By reducing the tendency of CNCs to aggregate through hydrogen bonding, silanization promotes a more homogeneous filler distribution. Compared to other fillers (such as inorganic ones, i.e., silica nanoparticles, clays or zeolites), silanization allows precise tuning of surface interactions, whereas inorganic fillers typically require more aggressive or energy-intensive treatments [41]. Moreover, the authors already implemented the modification of CNC in homogenous environment through silanization and subsequent grafting with bio-based polyols. The resulting modified filler was then employed as a reactive filler within PU foams [36], corresponding to a remarkable increase in the foam’s compressive strength (up to five times). The use of unmodified and modified CNCs in TPU nanocomposites have been illustrated by recent literature, enabling improvements mainly in thermal stability, mechanical properties, shape memory features [31,37,42,43], and hydrophobicity [30]. In particular, Mahadi et al. [31] demonstrated that the intercalation of unmodified CNCs in TPU matrices (in the range 0.5–2 wt.%) enabled higher mechanical strength. Unmodified CNCs were also employed by Fortunati et al. [30] in the synthesis of TPU nanocomposites through solvent casting routes; it was found that the water adsorption capability of the resulting nanocomposites was affected by CNC concentration, resulting in different distribution of HSs and SSs within TPU matrices. Additionally, silane-modified CNCs have been introduced by Sun et al. [44] as a reinforcing filler in TPU matrices, resulting in a significant enhancement in the tensile stress and toughness (of 72. 6% and 103.6%, respectively).

Based on these considerations, TPU/CNC nanocomposites can be regarded as emerging innovative materials where sustainability and functionality are combined, leading to promising systems for a wide range of applications. One such application is in the food packaging sector. Conventional packaging materials are still predominantly based on commodity plastic materials; however, these materials raise environmental concerns due to their fossil-derived origin and may pose potential safety issues associated with additives used to enhance their processability [45,46].

In this study, novel nanocomposites based on bio-based TPU reinforced with commercial silane-modified cellulose nanocrystals (Si−O−CNC) were developed and systematically characterized. TPU nanocomposites (TPU/Si−O−CNC) containing varying Si–O–CNC content (1–5 wt.%) were prepared via melt mixing followed by compression molding. The effect of Si−O−CNC incorporation into the TPU matrix was comprehensively evaluated through chemical (FTIR), thermal (TGA, DSC), mechanical (tensile testing), morphological (SEM), and rheological (frequency sweep) analyses, alongside a functional performance assessment in terms of water vapor permeability (WVP) and oxygen transmission rate (OTR). This work provides a platform for designing sustainable TPU-based nanocomposites, highlighting their potential for application in the food packaging sector.

## 2. Materials and Methods

### 2.1. Materials and Chemicals

Bio-based polyester based-TPU (namely ESTANE ECO 12T95, Lubrizol, Barcelona, Spain), with a density of 1.2 g/cm^3^, was purchased in the form of pellets. Silane-modified Cellulose Nano Crystals (Si−O−CNC) were kindly provided by Melodea Ltd. (Rehovot, Israel) as a powder with a concentration of 95.5%, a relative density of 1.5 g/cm^3^ and a particle core size of 5–20 nm in width and 100–500 nm in length (patent WO2017046798A1) [47].

All the materials were used as received.

### 2.2. TPU/Si−O−CNC Nanocomposites Preparation

For the preparation of TPU/Si−O−CNC nanocomposites, the two-step procedure reported by Russo et al. [26] was adopted: (i) TPU pellets were melted at 220 °C at 20 rpm for 2 min through a twin counter-rotating internal mixer (Rhemomix 600, Haake, Karlsruhe, Germany) connected to a control unit (Rheocord 9000, Haake, Karlsruhe, Germany), (ii) Si−O−CNC were then added and the speed increased to 50 rpm. Mixing was maintained for 8 min under the same conditions. Three nanocomposites with different Si−O−CNC concentrations (1, 3 and 5 wt.%, Table 1) were prepared. For comparison, pristine TPU was obtained by processing only TPU pellets. Nanocomposite films (thickness of 0.3 mm) were subsequently obtained through compression molding at 225 °C and 30 bar (P300P, Collin, Maitenbeth, Germany).

### 2.3. TPU/Si−O−CNC Nanocomposite Characterization

#### 2.3.1. Chemical/Structural Analysis

The chemical structure of Si−O−CNC, pristine TPU, TPU/Si−O−CNC nanocomposites was analyzed by Attenuated Total Reflectance Fourier Transform Infrared (ATR-FTIR) spectroscopy through FTIR Perkin Elmer Spectrum 100 series spectrophotometer (Waltham, MA, USA). Spectra were acquired at room temperature in the range of 4000 cm^−1^ to 650 cm^−1^ wavenumbers with a resolution of 4 cm^−1^ using 16 scans. Deconvolution of the peaks at 1700 cm^−1^ and 1730 cm^−1^ corresponding to hydrogen-bonded and free carboxylic groups of TPU hard segments, respectively, was performed through Lorentz function using Origin software (v. 269 16, Origin Lab, Northampton, MA, USA). Specifically, Carbonyl H-bond index (denoted as *R*) [38,48], was estimated according to Equation (1):

(1) $$R=A_{1700}/A_{1730}$$

where *A*_1700_ and *A*_1730_ are the computed areas of the above-mentioned peaks (expressed in arbitrary units, A.U.).

#### 2.3.2. Morphological Analysis

The morphological characterization of Si−O−CNC, pristine TPU and TPU/Si−O−CNC nanocomposites was assessed by Scanning Electron Microscopy (SEM, FEI Quanta 200 Field-Emission Gun, FEG, Scanning Electron Microscope, Hillsboro, OR, USA) under a vacuum with an accelerating voltage of 10–30 kV. Before being analyzed, samples were coated with Au/Pd alloy layer through sputtering (Emithech K575X, Quorum Technologies, Laughton, East Sussex, UK).

#### 2.3.3. Thermal Analysis

The thermal degradation of Si−O−CNC, pristine TPU and TPU/Si–O–CNC nanocomposites was evaluated by thermogravimetric analysis (TGA). Tests were carried out on a TGA Q5000 (TA Instruments, New Castle, DE, USA) over a temperature range from 30 °C to 600 °C at 10 °C/min under N_2_ atmosphere (flow rate 50 mL/min).

Differential Scanning Calorimetry (DSC) was carried out to evaluate the melting temperature (*T*_m_) and glass transition (*T*_g_) temperature of the pristine TPU and TPU nanocomposites. Then, 7 mg samples were loaded into a standard aluminum pan and analyzed by a Discovery DSC 2500 (TA Instruments, New Castle, DE, USA) in N_2_ atmosphere (with a balance purge flow of 10 mL/min and a sample purge flow of 20 mL/min). A heating-cooling–heating cycle was employed in a temperature range from −90 °C to 250 °C with a constant-temperature ramp of 10 °C/min. The first heating scan was meant to cancel the thermal history of the materials; hence, the assessment of thermal properties was carried out at the second heating scan.

#### 2.3.4. Rheological Analysis

The rheological analysis was performed on TPU/Si−O−CNC nanocomposites by using a stress controlled-rotational rheometer (RheoScope MARS II, HAAKE, Karlsruhe, Germany) equipped with 20 mm parallel plates. The tests were conducted from 200 °C to 220 °C under N_2_ atmosphere, using a gap thickness of 0.2 mm. Frequency sweep tests were carried out from 0.01 Hz to 100 Hz with a fixed strain, ζ, of 0.1% in order to operate in the linear viscoelastic region [49].

#### 2.3.5. Mechanical Analysis

The tensile tests were performed at room temperature according to ASTM standard D882-18 [50] by using a CMT 4304 SANS Testing Machine (SANS, Shenzhen, China) equipped with a 2.5 kN load cell. Based on the stress vs. elongation curve, the Young’s modulus *(E*, MPa), stress (*σ_b_*, MPa) and elongation at break (*ε_b_*, mm/mm) were calculated [51]. The stress and strain at break were calculated at the last point of the stress–strain curve before failure, whereas *E* was evaluated based on the first linear region. For each sample, results were expressed as the average and standard deviation (SD) of five independent measurements.

#### 2.3.6. Barrier Properties Analysis

The Water Vapor Permeability (WVP) and Oxygen Transmission Rate (OTR) of pristine TPU and TPU/Si−O−CNC nanocomposites were assessed by means of Permatran-W^®^ 3/34 and Oxtran 2/22 permeation analyzers (Ametek Mocon, Brooklyn Park, MN, USA), respectively. Tests were performed at least in triplicate in controlled conditions (T = 25 °C, relative humidity, RH = 50%) for 48 h. For OTR measurements, the permeant oxygen concentration was set to 100%. Specimens with a surface area of 5 cm^2^ were prepared for testing.

#### 2.3.7. Wettability Analysis

The wettability of the pristine TPU and TPU/Si−O−CNC nanocomposites was evaluated by contact angle measurements of deionized water (i.e., Water Contact Angle, WCA) by the sessile drop method [52] using an OCA 20 (Dataphysics, Filderstadt, Germany) goniometer. Data were collected with SCA 202 software (version 3.4.3 build 76, Filderstadt, Baden-Württemberg, Germany). Static contact angles (at the equilibrium) were measured for 1 μL volume droplets, expressed as an average of left and right WCA. Measurements were taken at 10 different locations for each condition, and the average value was reported with the SD.

## 3. Results and Discussion

### 3.1. Chemical/Structural Analysis

**Figure 1:**
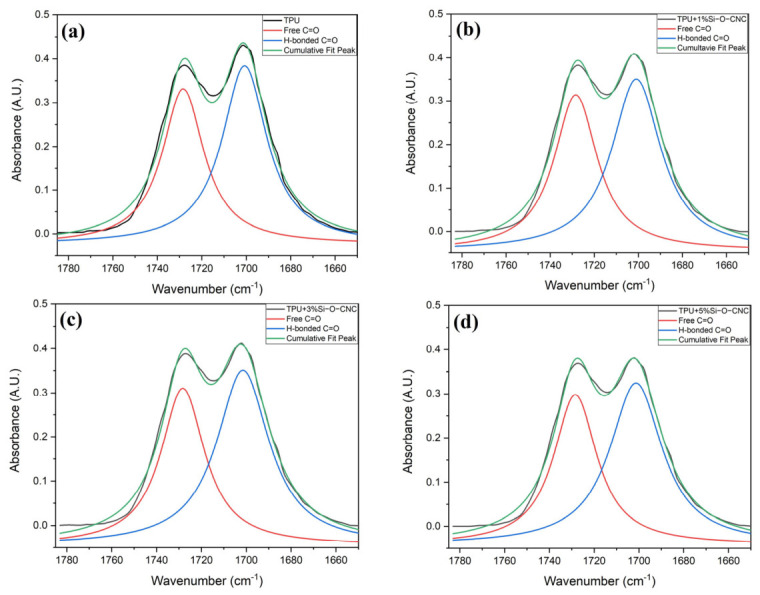
FTIR spectra deconvolution of 1700 cm^−1^ and 1730 cm^−1^ peaks for (**a**) pristine TPU, (**b**) TPU + 1%Si−O−CNC, (**c**) TPU + 3%Si−O−CNC and (**d**) TPU + 5%Si−O−CNC.

FTIR spectra of pristine TPU, Si−O−CNC, and TPU/Si−O−CNC nanocomposites are presented in Figure S1 of the Supplementary Materials, whereas the main peak assignments are summarized in Table S1. Figure 1a–d show the deconvolution of the spectral region associated with free and hydrogen-bonded carbonyl groups (C=O), as well as urea-related carbonyl signals, for pristine TPU and TPU/Si−O−CNC nanocomposites [26,53,54,55,56,57]. Nevertheless, assessing the chemical modification of CNCs was outside the scope of this work.

The results indicate that the incorporation of Si−O−CNC led to notable changes in the relative band areas of the free-C=O (~1730 cm^−1^) and H-bonded-C=O (~1700 cm^−1^) groups, which belong to the HS. Specifically, an increase in *R* value (Table 2), was observed, suggesting enhanced hydrogen-bonding interactions, likely between the hydroxyl (-OH) groups of Si−O−CNC and the carbonyl groups in TPU [58]. These changes indicate that the presence of Si−O−CNC may affect the hydrogen-bonding environment and, consequently, the microphase organization of TPU. Furthermore, the presence of Si−O−CNC also may indicate the possible formation of urea-related structures within the nanocomposite structure, as shown by the appearance of its characteristic absorption band at 1637cm^−1^ [59,60].

### 3.2. Morphological Analysis

**Figure 2:**
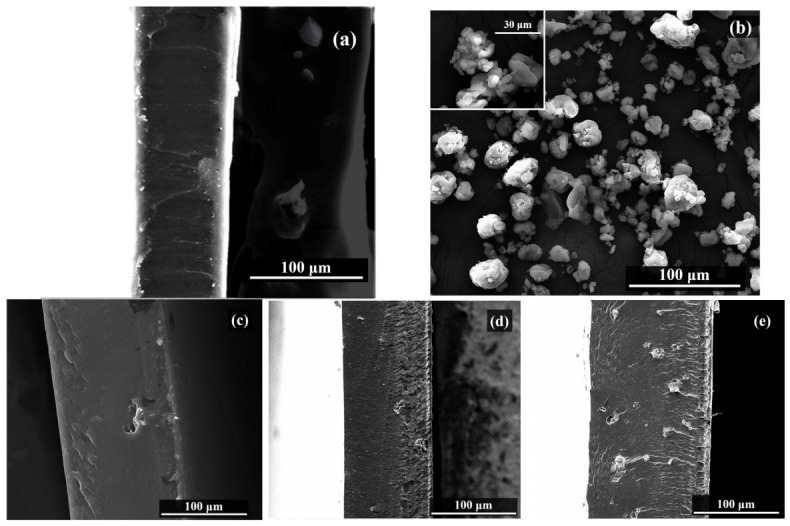
Scanning Electron Microscopy (SEM) images of (**a**) pristine TPU, (**b**) Si−O−CNC (inset Magnification 4000×, scale bar = 30 μm), (**c**) TPU + 1%Si−O−CNC, (**d**) TPU + 3%Si−O−CNC, (**e**) TPU + 5%Si−O−CNC nanocomposites. Magnification 1000×, scale bar = 100 μm.

Cross-section SEM images of pristine TPU and TPU-based nanocomposites are displayed in Figure 2a–e. Images of Si−O−CNC are reported in Figure 2b. Pristine TPU exhibited a smooth surface (Figure 2a). In Si−O−CNC powder (Figure 2b), micron-sized sphere-like aggregates with an average diameter of 35.9 ± 10.4 μm were observed. These structures likely formed during silanization, followed by drying and storage, and do not represent the intrinsic rod-like nanoscale dimensions of individual CNCs. Surface hydrophobization introduces organosilane moieties that modify interparticle interactions, promoting aggregation into compact clusters rather than isolated nanocrystals [61,62,63,64].

The absence of these micron-sized clusters in the final TPU-based composites can be reasonably ascribed to the hot-mixing process, during which the applied shear stresses promote the disaggregation of CNC agglomerates and improve their dispersion within the TPU matrix. Higher-magnification SEM images acquired at 2000× are shown in Figure S2 of the Supplementary Materials to better assess the absence of evident agglomerates, particularly at low filler loading. In case of TPU + 1%Si−O−CNC (Figure 2c, with a thickness of about 130 μm), Si−O−CNCs were not particularly noticeable. At higher contents, Si−O−CNC tended to re-aggregate, as clearly observed in TPU + 5%Si–O–CNC (Figure 2e). This phase segregation was ascribed to the high filler content, which favored filler-filler interactions over filler-matrix interactions [65]. Based on these results, although TPU + 3%Si−O−CNC (Figure 2d) may still appear relatively well dispersed, the most homogeneous morphology was obtained at 1 wt.% filler.

### 3.3. Thermal Analysis

The thermal properties of the resulting TPU/Si−O−CNC nanocomposites were investigated through TGA and DSC analyses. TGA is a useful tool to accurately assess the influence of the filler on TPU thermal degradation behavior. Segmented PUs typically exhibit distinct degradation temperatures within their hard segments (HSs) and soft segments (SSs). This behavior is particularly evident in the Derivative Thermogravimetric Analysis (DTGA) curves, which display two characteristic peaks corresponding to the maximum degradation temperatures of HSs and SSs.

**Figure 3:**
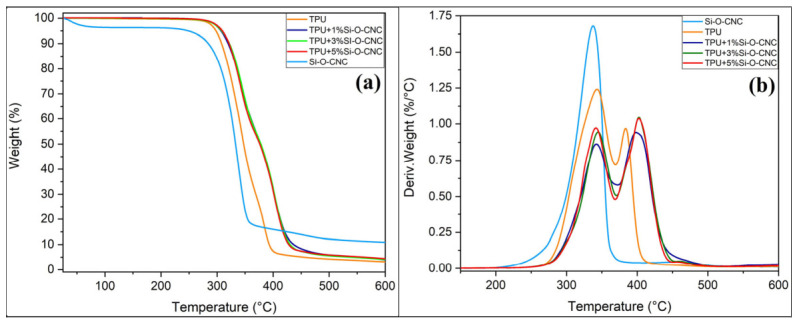
Thermogravimetric (**a**) and Derivative Thermogravimetric (**b**) curves of pristine TPU and TPU/Si−O−CNC nanocomposites.

TGA and DTGA curves of the pristine TPU, Si−O−CNC, and TPU-based nanocomposites are displayed in Figure 3a,b. Pristine TPU underwent a two-step degradation process, with the corresponding maximum degradation temperatures (*T*_max1_ and *T*_max,2_, Table 3). The first degradation stage is typically associated with the thermal decomposition of HSs, primarily involving urethane bond cleavage, leading to the release of diols, di-isocyanates, and low-molecular-weight species. The second degradation stage corresponds to the breakdown of polyols within the SSs [66,67,68,69].

For Si−O−CNC, a slight initial weight loss occurred below 100 °C due to the evaporation of water weakly bound to cellulose surfaces. The main degradation step was observed at approximately 337 °C and was attributed to concurrent phenomena such as depolymerization, glycosidic ring degradation, and breakdown of the crystalline regions [70,71]. The resulting nanocomposites exhibited enhanced thermal stability compared to pristine TPU. Specifically, the first degradation step (*T*_max1_) remained unaffected by the presence of the filler, whereas a notable shift was observed for *T*_max2_, which increased by up to 20 °C compared to *T*_max2_ of pristine TPU. The selective shift in *T*_max2_ to higher temperatures may be related to the hydrophobic nature of the silane-modified CNCs. As reported by Ristić et al. [72], the degree of interaction between filler and TPU segments depends on hydrophobicity: hydrophilic fillers preferentially interact with HSs, whereas hydrophobic fillers tend to associate with SSs. The higher thermal stability of the nanocomposites compared to pristine TPU was further evidenced by an increase of approximately 10 °C in *T*_5%_ (temperature at 5% weight loss) and about 30 °C in *T*_50%_ (temperature at 50% weight loss), regardless of filler concentration (Table 3). Typically, this improvement is ascribed to interactions between the hydroxyl groups of CNCs and the C=O, N-H, and C–O–C groups of TPU [33].

Upon incorporation of Si–O–CNC, the overall reduction in the degradation rate, accompanied by a change and even inversion in the relative height of the DTGA peaks, was noticed (Figure 3b). This behavior can be attributed to the incorporation of CNCs, which disrupt the strong intermolecular interactions within the hard segments (HSs) and reduce the size, cohesion, and structural integrity of the hard domains. As a result, the contribution of the hard phase was diminished, while the relative influence of the soft segments (SSs) became more pronounced. The weakening and partial disintegration of the hard domains reduce the degree of microphase separation between the HSs and SSs, promoting greater intermixing between the two phase, resulting in greater mobility of the SSs and facilitating more efficient heat transfer throughout the PU matrix [73]. In Table 3 the residual masses at 600 °C and the onset degradation temperatures for the two degradation stages (*T*_ons max1_ and *T*_ons max2_**)** are also reported. Overall, the resulting nanocomposites exhibited a greater residual mass than pristine TPU, with the amount of residue increasing as the filler content increased.

**Figure 4:**
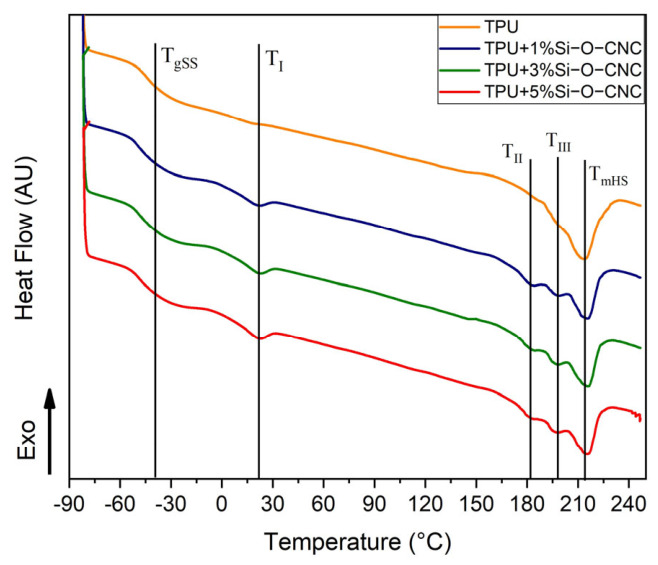
Differential Scanning Calorimetry (DSC) curves of pristine TPU and TPU/Si−O−CNC nanocomposites during the second heating scan.

Figure 4 displays the second heating scan of the DSC curves of pristine TPU and TPU-based-nanocomposites.

Pristine TPU exhibited a glass transition temperature (*T*_gSS_) at −44.0 °C, associated with the SSs, and a melting temperature (*T*_mHS_) at 213.8 °C, corresponding to the hard domains, which represent the crystalline domain of TPU (Table 4) [43]. This indicates that TPU remained in a rubbery state at room temperature. Incorporating Si−O−CNC into the TPU matrix slightly lowered *T*_gSS_ (−49.9 °C), delaying embrittlement and allowing the nanocomposite to maintain softness at lower temperatures. This downward shift in *T*_g_ suggested increased chain mobility, consistent with observations from TGA. The endothermic peak of pristine TPU between 180 and 220 °C displayed two shoulders near 184 °C and 198 °C, attributed to disordered SSs melting earlier than ordered ones [74,75]. *T*_mHS_ remained essentially unchanged in the nanocomposites, indicating that the filler did not affect the main crystalline melting point. Notably, TPU/Si−O−CNC nanocomposites exhibited three endothermic peaks. Specifically, the shoulders (observed in pristine TPU) evolved into two separate peaks at ~182 °C (*T*_II_) and ~198 °C (*T*_III_), while an additional weak low-temperature endothermic event (*T*_I_), which was not clearly detectable in pristine TPU, appeared near 23 °C [76]. This signal may be attributed to a soft-phase-related thermal transition, possibly involving the melting of imperfectly ordered soft-segment domains and relaxation and reorganization phenomena occurring at the soft/hard segment interface [77,78]. Specifically, these new peaks (*T*_II_ and *T*_III_) may arise from hydrogen bonding between Si−O−CNC and HSs, promoting the formation of smaller, less ordered hard domains that melt before *T*_mHS_ [31]. This result is consistent with the FTIR analysis, which indicated that the increase in the hydrogen-bonding coefficient promotes interactions between the Si–O–CNC groups and the carbonyl functionalities of HSs, leading to the formation of smaller hard domains. Therefore, the melting enthalpy (ΔH_m_) of TPU nanocomposites decreased with respect to that of pristine TPU.

### 3.4. Rheological Analysis

**Figure 5:**
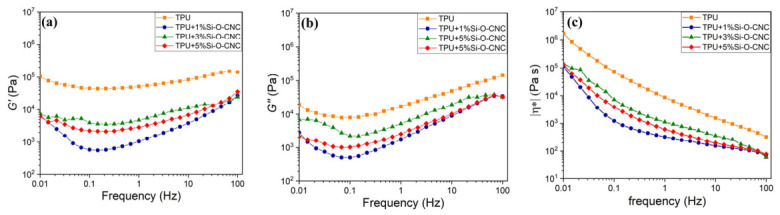
(**a**) *G*′, (**b**) *G*″ and (**c**) |η*| as a function of frequency of pristine TPU and TPU/Si−O−CNC nanocomposites.

Figure 5a–c illustrate the frequency dependence of the storage modulus (*G*′, Pa), loss modulus (*G*″, Pa), and complex viscosity (|η*|, Pa·s) for pristine TPU and TPU/Si−O−CNC nanocomposites. Pristine TPU exhibited the typical rheological behavior of a phase-segregated PU. *G*″ remained higher than *G*′ over the entire frequency range, indicating the dominance of viscous relaxation phenomena associated with SSs mobility. The pronounced decrease in both moduli at low frequencies reflected long-time relaxation processes, such as chain disentanglement and reversible rearrangement of hydrogen-bonded hard domains [79,80]. The pronounced shear thinning in |η*| further indicated a broad distribution of relaxation times, typical of systems with dynamic hard-domain connectivity. The addition of 1 wt.% Si–O–CNC led to the most significant modification of melt behaviour. Both *G*′ and *G*″ decreased, with *G*″ showing the strongest reduction, indicating a decrease in viscous dissipation mechanisms. This response may be related to the relatively good dispersion of Si–O–CNC, enabling the filler to disrupt TPU micro-phase organization. Interactions between Si−O−CNC surface groups and the HSs of the TPU, as highlighted by FTIR analysis, may modify the hydrogen-bonding network and introduce additional interfacial constraints, leading to reduced SSs mobility and a narrower relaxation spectrum. These trends are consistent with previous observations in CNCs-reinforced PU systems [79,81].

At 3 wt.% Si−O−CNC, such effects became less pronounced. Although the overall shapes of the *G*′ and *G*″ curves remained similar to those observed at 1 wt.%, the reductions in *G*″ and |η*| were noticeably smaller. In addition, a slight increase in *G*′ at intermediate frequencies suggested the onset of localized reinforcement effects. The weaker impact on the relaxation behavior may indicate a reduction in effective polymer-filler interactions, likely resulting from the initial aggregation of Si−O−CNC particles, which decreases the available surface area for interaction with the TPU matrix [44,82,83]. At 5 wt.%, the system appeared to reach a saturation regime. Both *G*′ and *G*″ increased nearly in parallel while maintaining *G*″ *> G*′ across the entire frequency range, confirming that the melt retained its predominantly viscous character. Furthermore, the |η*| curve almost completely overlapped with that of the 3 wt.% nanocomposite, indicating that additional filler loading did not markedly affect the relaxation dynamics. SEM observations revealed extensive filler aggregation at this concentration, consistent with previous reports on CNC- and silica-reinforced PU and polysaccharide composites [84,85,86]. Under these conditions, the filler may behave largely as an inert particulate phase, exerting only a limited influence on polymer chain mobility. Overall, the rheological results suggest that the effect of Si−O−CNC on TPU melt dynamics was governed primarily by dispersion quality rather than filler concentration alone. In low amounts, well-dispersed Si−O−CNC may interact more effectively with the polymer matrix, disrupting hydrogen-bonding networks, constraining soft-segment dynamics, and modifying the relaxation spectrum. In contrast, aggregation at higher contents reduced the effective interfacial area available for polymer–filler interactions, thereby diminishing the ability of Si−O−CNC to alter the viscoelastic response of the TPU matrix.

### 3.5. Mechanical Analysis

**Figure 6:**
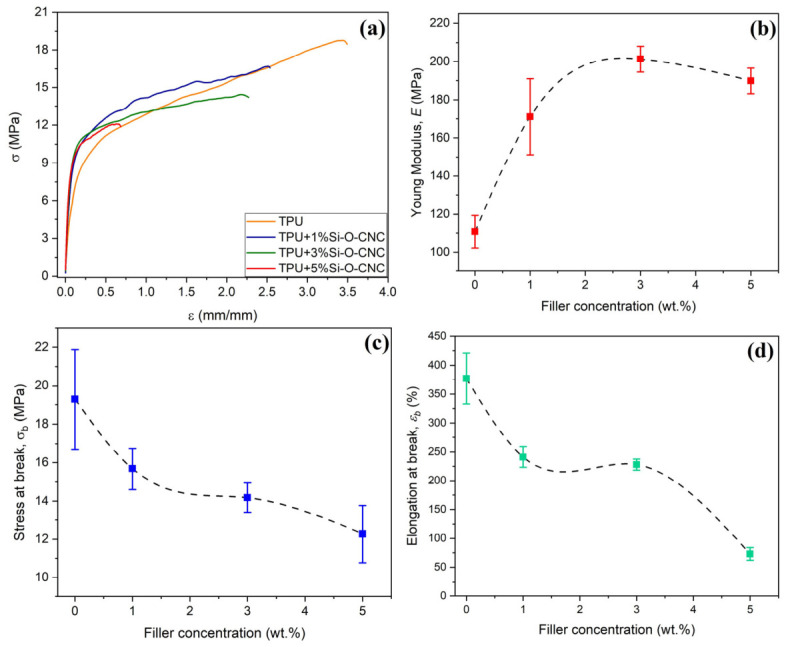
(**a**) Stress–strain curves, (**b**) Young’s modulus, *E*, (**c**), stress at break, $\sigma_{b}$, (**d**) elongation at break, $\varepsilon_{b}$ of pristine TPU and TPU/Si−O−CNC nanocomposites. Data are expressed as the average and SD of five independent measurements.

The mechanical behavior of pristine TPU and TPU-based nanocomposites was evaluated at ambient temperature through uniaxial tensile tests to failure. The corresponding stress–strain curves are presented in Figure 6a. The Young’s modulus (*E*), stress at break (*σ_b_*), and elongation at break (*ε_b_*) are summarized in Figure 6b–d.

Pristine TPU displayed the characteristic deformation profile of thermoplastic polyurethane elastomers, consisting of an initial elastic stage, followed by plastic deformation associated with the stretching and disentanglement of the soft domains, and finally by the progressive rupture of the hard domains. The final stress upturn prior to fracture was consistent with strain-induced crystallization of the oriented SSs and with structural rearrangements within the hard phase, which together contributed to strain hardening [87,88].

The incorporation of Si–O–CNC altered the deformation behavior of TPU. A clear increase in stiffness was observed for all investigated conditions, resulting in higher *E* values (Figure 6b). The maximum stiffness was achieved at 3 wt.%, where *E* increased to 201.3 ± 6.74 MPa, approximately twice that of pristine TPU (110.8 ± 8.56 MPa). At higher filler contents, the slight decrease in modulus was consistent with reduced dispersion quality and the formation of Si–O–CNC agglomerates. Nevertheless, a significant portion of the filler remained well dispersed, allowing the nanocomposites to retain modulus values above those of the pristine TPU.

Conversely, both $\sigma_{b}$ and $\varepsilon_{b}$ decreased at increased filler contents (Figure 6c,d), indicating reduced ductility. $\sigma_{b}$ progressively declined from approximately 20 MPa for pristine TPU to ~12 MPa, while $\varepsilon_{b}$ dropped more sharply from ~400% to below 60% at 5 wt.%. The substantial decrease, already observed at 1 wt.%, suggested that even small additions of filler were sufficient to modify the structural integrity of the matrix. The enhancement in stiffness arose from two synergistic microstructural mechanisms. First, the incorporation of Si−O−CNC strengthened the fillermatrix interfacial interactions, as corroborated by FTIR analysis. Enhanced adhesion at the polymer-filler interface promotes more efficient stress transfer from the TPU matrix to the CNC phase while simultaneously constraining macromolecular mobility. Together, these effects contribute to an increase in *E* [30,44,86].

Second, the FTIR analysis may also indicated the formation of urea-related structures, corresponding to an increase in HSs content [59,89], thus promoting more extensive aggregation of the hard domains [90,91,92]. The resulting reorganization of the microphase-separated morphology yielded a more rigid physical crosslinking network, thereby restricting chain mobility and contributing to the microstructural stiffening effect [44,93]. The combined influence of reinforced interfacial bonding and hard-domain evolution reduced also the material’s ability to undergo plastic deformation, leading to lower stress and elongation at break and to an earlier onset of structural failure compared to the pristine TPU [7,94]. In summary, these results confirmed that the mechanical performance of TPU/Si−O−CNC nanocomposites is primarily governed by the content of incorporated filler. Specifically, concentrations in the range of 1 wt.% may offer an optimal balance between increased stiffness and suitable ductility.

### 3.6. Barrier Properties Analysis

**Figure 7:**
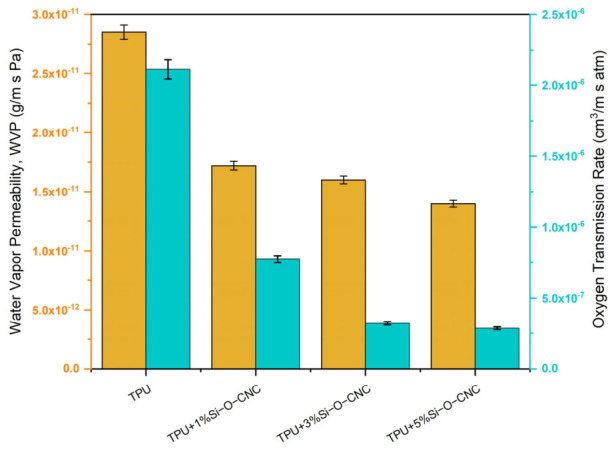
Water vapor permeability (WVP) (yellow bars)and oxygen transmission rate (OTR) (teal bars) of pristine TPU and TPU/Si−O−CNC nanocomposites. Data are expressed as the average and SD of at least three independent measurements.

The water vapor permeability (WVP) and oxygen transmission rate (OTR) results are reported in Figure 7. The addition of Si−O−CNC resulted in a decrease in both WVP and OTR, both decreasing by approximately 50% compared with pristine TPU. Overall, a clear decreasing trend was observed under all the examined conditions. This effect may be attributed to the embedding of a hydrophobic filler in TPU, which contributes to the reduction in the WVP. In addition, Si−O−CNC acted as a physical barrier, promoting the formation of a tortuous pathway for water molecules inside the matrix, resulting in the decrease in OTR [95,96,97,98]. Moreover, the changes in hard domains dimension, as highlighted by DSC analysis, may further hinder permeant diffusion through the films. A significant decrease in WVP and OTR was observed at 1 wt.% Si−O−CNC. However, further increases in filler content resulted in progressively smaller reductions in permeability, indicating a gradual saturation of the barrier-enhancement effect. Based on these results, TPU+1%Si−O−CNC nanocomposite appeared to be particularly promising in food packaging applications where enhanced barrier performance is required. In particular, its reduced oxygen and water vapor permeability could be beneficial for fresh meat packaging under modified-atmosphere packaging (MAP) conditions, where maintaining a controlled gaseous environment is essential for preserving product quality and extending shelf life [99].

### 3.7. Wettability Analysis

**Figure 8:**
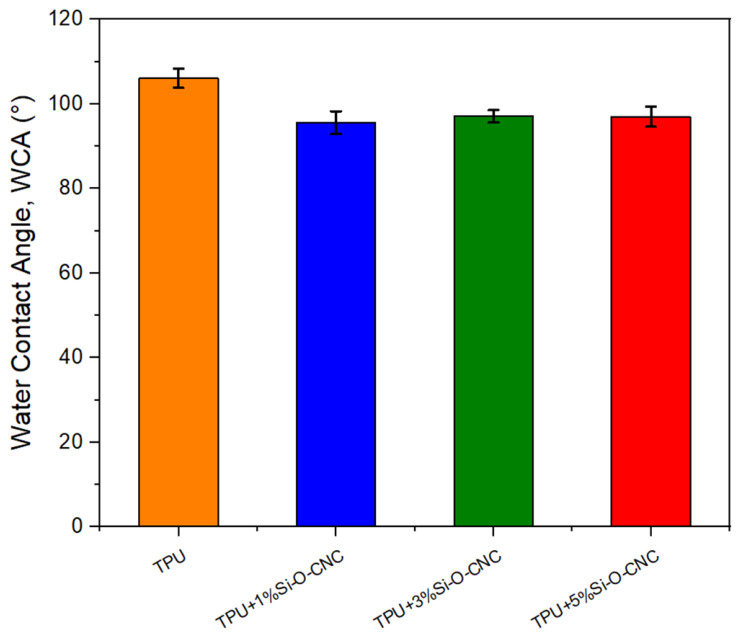
Water contact angle values for pristine TPU and TPU/Si−O−CNC samples. Data are expressed as the average and SD of three independent measurements.

The wettability of the TPU/Si−O−CNC nanocomposites, expressed in terms of the static water contact angle (WCA), is shown in Figure 8. Pristine TPU exhibited hydrophobic behavior, with a contact angle of 106.1°± 2.3°, in agreement with previous literature [28,100]. Upon the addition of Si−O−CNC, a progressive decrease in WCA was observed, reaching a minimum value of 97.0° ± 2.4° at the highest filler content. Nevertheless, all formulations remained within the hydrophobic regime, as the threshold between hydrophobic and hydrophilic behavior is conventionally set at contact angle (CA) equal to 90° [101]. Although the observed reduction in WCA might initially appear inconsistent with the concomitant decrease in WVP, these measurements probe fundamentally distinct phenomena. Specifically, WCA reflects the wettability and surface energy characteristics of the material, whereas WVP is governed by the mechanisms of water-vapor sorption, diffusion, and transport through the bulk polymer matrix. From this perspective, Si−O−CNC may contribute to reducing bulk water transport by interacting with the TPU matrix and creating a more tortuous pathway for diffusing water molecules. Concurrently, a minor fraction of filler particles might be exposed at the nanocomposite film surface rather than being fully embedded within the TPU matrix, thereby locally reducing surface hydrophobicity [102]. This interpretation is also consistent with the concentration-dependent morphology observed by SEM and with the rheological results. At low filler content, better Si−O−CNC dispersion appears to favor polymer-filler interactions and bulk barrier improvement. Conversely, at higher filler content, the aggregation of Si−O−CNC may favour the formation or exposure of localized hydrophilic domains that further lowered WCA. Therefore, TPU + 1%Si−O−CNC nanocomposite displayed the best balance between surface properties and bulk barrier performance.

## 4. Conclusions

In this work, commercially supplied silane-modified cellulose nanocrystals (Si−O−CNC) were successfully employed as a sustainable nanofiller to fabricate bio-based TPU nanocomposites with enhanced mechanical and functional properties (water vapor permeability and oxygen transmission rate). However, it should be noted that the investigation of the chemical modification of CNCs through silanization was beyond the aim of this work. The nanocomposites, containing varying Si−O−CNC content (1–5 wt.%), were prepared via melt mixing followed by compression molding, and were extensively characterized. The addition of Si−O−CNC induced a significant increase (up to 8%) in the hydrogen-bonding interactions within the TPU matrix, as quantitatively estimated through the carbonyl hydrogen-bonding index (*R*), obtained via FTIR analysis. This corresponds to the enhancement of thermal degradation stability compared to pristine TPU. This improvement was accompanied by the formation of smaller hard domains characterized by a short-range order. Moreover, the incorporation of Si−O−CNC promoted a noticeable increase in material stiffness, yielding a two-fold increase in the Young’s modulus (*E*) at 1 wt.% filler content. This reinforcement may be associated with a synergistic effect involving enhanced hydrogen bonding and possible formation of urea moieties, which increased the overall hard-segment content. Overall, the resulting nanocomposites exhibited reduced water vapor permeability and oxygen transmission rate compared to pristine TPU across all investigated compositions, while remaining in the hydrophobic regime.

Overall, this study provides valuable insights into the design of sustainable TPU-based nanocomposites, demonstrating that the incorporation of silane-modified CNCs yielded high-performance materials with promising potential for advanced food packaging applications, such as the preservation of fresh meat in modified-atmosphere packaging (MAP). Future studies will focus on the assessment in terms of microbial growth inhibition, physicochemical quality preservation (weight loss, color stability, lipid oxidation or sensory quality), stability in time, and shelf-life extension of the fresh meat products of the resulting nanocomposites.

## Figures and Tables

**Table 1:** Composition of TPU/Si−O−CNC nanocomposites.

Sample	TPU (wt.%)	Si−O−CNC (wt.%)
TPU	100	0
TPU + 1%Si−O−CNC	99	1
TPU + 3%Si−O−CNC	97	3
TPU + 5%Si−O−CNC	95	5

**Table 2:** Carbonyl hydrogen bond index (*R*) of pristine TPU and TPU-based nanocomposites.

Sample	*A*_1700_ (A.U.)	*A*_1730_ (A.U.)	*R* (-)
TPU	15.8	12.8	1.23
TPU + 1%Si−O−CNC	17	13.5	1.26
TPU + 3%Si−O−CNC	18	13.2	1.36
TPU + 5%Si−O−CNC	16.5	12.6	1.31

**Table 3:** Thermal properties of pristine TPU, Si−O−CNC and TPU-based nanocomposites obtained from Thermogravimetric analysis (TGA) and Derivative Thermogravimetric analysis (DTGA).

Sample	*T*_5%_ (°C)	*T*_50%_ (°C)	*T*_ons max1_ (°C)	*T*_max1_ (°C)	*T*_ons max2_ (°C)	*T*_max2_ (°C)	Residual Weight at 600 °C (wt.%)
Si−O−CNC	249	333	305	337	//	//	10.8
TPU	298	347	307	343	377	383	3.2
TPU + 1%Si−O−CNC	307	377	312	342	385	398	3.9
TPU + 3%Si−O−CNC	308	378	316	345	388	402	4.1
TPU + 5%Si−O−CNC	309	376	314	341	387	402	4.4

**Table 4:** Thermal properties of pristine TPU and TPU/Si−O−CNC nanocomposites obtained from Differential Scanning Calorimetry (DSC) analysis.

		Endothermal Peaks				
Sample	*T*_gSS_ (°C)	*T*_I_ (°C)	*T*_II_ (°C)	*T*_III_ (°C)	*T*_mHS_ (°C)	ΔH_m_ (J/g)
TPU	−44.0	n.d.	n.d.	n.d.	213.8	16.9
TPU + 1%Si−O−CNC	−49.0	23.0	182.4	198.5	215.9	13.2
TPU + 3%Si−O−CNC	−49.9	22.9	183.1	199.0	215.6	13.8
TPU + 5%Si−O−CNC	−48.7	22.8	182.7	198.2	215.5	13.5

## Data Availability

The original contributions presented in this study are included in this article/Supplementary Materials. Further inquiries can be directed to the corresponding authors.

## Supplementary Materials

The following supporting information can be downloaded at: https://www.mdpi.com/article/10.3390/polym18131665/s1, Figure S1: FTIR spectra of pristine TPU, Si−O−CNC and TPU/Si−O−CNC nanocomposites; Figure S2: Scanning Electron Microscopy (SEM) images of (a) TPU+1%Si−O−CNC, (b) TPU+3%Si−O−CNC, (c) TPU+5%Si−O−CNC at higher magnification; Table S1: Vibrational assignment of the FTIR spectra for pristine TPU and TPU/Si−O−CNC nanocomposites.

## Abbreviations

The following abbreviations are used in this manuscript:

Ag	Silver
ATR-FTIR	Attenuated Total Reflectance Fourier Transform Infra-Red.
Au	Gold
*A*_1700_	Area of the peak at 1700 cm^−1^
*A*_1730_	Area of the peak at 1730 cm^−1^
CA	Contact Angle
CNC	Cellulose Nano Crystals
Cu	Copper
DSC	Differential Scanning Calorimetry
DTGA	Derivative Thermogravimetric Analysis
*E*	Young’s Modulus
*G*′	Storage Modulus
*G*″	Loss Modulus
HSs	Hard Segments
MAP	Modified Atmosphere Packaging
N_2_	Nitrogen
OTR	Oxygen Transmission Rate
Pd	Palladium
PU	Polyurethane
*R*	Carbonyl H-Bond Index
RH	Relative Humidity
SD	Standard Deviation
SEM	Scanning Electron Microscopy
Si−O−CNC	Silane-Modified Cellulose Nanocrystals
SSs	Soft Segments
*T*	Temperature
*T_g_*	Glass Transition Temperature
T*_gSS_*	Glass Transition Temperature associated with SS
TGA	Thermogravimetric Analysis
TiO_2_	Titanium Oxide
*T_ons max_*_1_	Onset of degradation temperature of the first degradation stage
*T_ons max_*_2_	Onset of degradation temperature of the second degradation stage
*T_m_*	Melting Temperature
*T_mHS_*	Melting Temperature associated with hard segments
*T*_max 1_	Maximum Degradation rate at first degradation step
*T*_max2_	Maximum Degradation rate at second degradation step
*T*_5%_	Temperature at 5% weight loss
*T*_50%_	Temperature at 50% weight loss
$T_{I}$	Temperature at the peak of the melting of the soft segments
$T_{Ii}$	Temperature at the first peak of the melting of the hard segments
$T_{III}$	Temperature at the second peak of the melting of the hard segments
TPU	Thermoplastic Polyurethane
TPU/Si−O−CNC	Thermoplastic Polyurethane nanocomposites reinforced with Silane-Modified Cellulose Nanocrystals
WCA	Water Contact Angle
WVP	Water Vapor Permeability
ZnO	Zinc Oxide
ΔH_m_	Enthalpy of melting
$\varepsilon_{b}$	Elongation at Break
*σ_b_*	Stress at Break
ζ	Strain
|*η**|	Complex viscosity
