# Supplementary material for: Design and Development of High-Performance Bio-Based Thermoplastic Polyurethane (TPU) Nanocomposites Enabled by Silane-Modified Nanocellulose
Source: Polymers (Basel). 2026 Jul 5;18(13):1665. doi: 10.3390/polym18131665 (PMC13363711; doi:10.3390/polym18131665)
Supplement: Supplementary file 1 [file polymers-18-01665-s001.zip › polymers-4368652-supplementary.pdf]

polymers

MDPI

Article

Design and Development of High-Performance Bio-Based Thermoplastic Polyurethane (TPU) Nanocomposites Enabled by Silane-Modified Nanocellulose

Nello Russo [1,†], Federica Recupido [2,†], Loredana Tammaro [3], Maria Oliviero [1,*], Barbara Liguori [2], Roberta Marzella [1], Letizia Verdolotti [1,*] and Giuseppe Cesare Lama [1]

[1] Institute of Polymers, Composites and Biomaterials, National Research Council of Italy (IPCB-CNR), Piazzale E. Fermi, 1, 80055 Portici, Italy; nellorusso@cnr.it (N.R.); roberta.marzella@cnr.it (R.M.); giuseppecesare.lama@cnr.it (G.C.L.)

[2] Department of Chemical, Materials and Industrial Production Engineering (DiCMaPI), University of Naples Federico II, Piazzale V. Tecchio 80, 80125 Naples, Italy; federica.recupido@cnr.it (F.R.); bliguori@unina.it (B.L.)

[3] Laboratory Smart Components and Systems for Sustainable Manufacturing, Department for Sustainability, Division Technologies and Materials for Sustainable Manufacturing Industry, Italian National Agency for New Technologies, Energy and Sustainable Economic Development (CMS-TIMAS-SSPT-ENEA) Piazzale E. Fermi 1, 80055 Portici, Italy; loredana.tammaro@enea.it

* Correspondence: maria.oliviero@cnr.it (M.O.), letizia.verdolotti@cnr.it (L.V.)

† These authors contributed equally to this work.

Supplementary Materials

The spectra of the pristine TPU and TPU/Si−O−CNC nanocomposites are reported in **Figure S1.** The main assignments are summarized in **Table S1**.

The spectrum of the pristine TPU revealed distinct absorption bands at 3324, 2956, and 2871 cm^{-1}, corresponding to the symmetric stretching vibrations of the –NH and CH_2 groups. The absence of any detectable band within the 2500-2000 cm^{-1} region, typically associated with residual isocyanate (–N=C=O) functional groups, confirmed that the material underwent complete polymerization. Absorption peaks, observed at 1728 cm^{-1} and 1700 cm^{-1}, were assigned to the stretching modes of free and hydrogen-bonded carbonyl (C=O) groups of the hard segments, respectively. The combined stretching vibrations of amide II (C–N + δN–H) and amide III (C–N + C=O) of the urethane linkage were identified at 1529 cm^{-1} and 1254 cm^{-1}. Furthermore, the bands located at 1478, 1458, 1413, and 1360 cm^{-1} were attributed to the bending vibrations of the –CH_2 moieties. Finally, the absorption features at 1075 cm^{-1} and 770 cm^{-1}, corresponding to the vibrations of C−O−C functional groups of polyols, respectively, provided information about the chemical nature of the soft segment. The Si−O−CNC spectrum (**Figure S1**) exhibited characteristic absorption bands at 3334 cm^{-1} and 3293 cm^{-1}, corresponding to the symmetric stretching of hydroxyl (–OH) groups. Peaks observed at 2902 cm^{-1}, 1428 cm^{-1}, and 1370 cm^{-1} were assigned to the symmetric stretching of –CH groups and to the asymmetric and symmetric bending vibrations of C–H bonds, respectively. The absorption band at 1642 cm^{-1}, associated with the O-H stretching vibration, indicated the presence of adsorbed moisture within the nanocellulose. Furthermore, bands at 1160 cm^{-1}, 1053 cm^{-1}, and 897 cm^{-1} were attributed to the asymmetric stretching and bending modes of C−O−C glycosidic linkages and to the C−O−C vibrations of the pyranose ring, respectively. Finally, the absorption features at 1053 and 1030 cm^{-1} were assigned to the presence of Si–O–C and Si–O–Si linkages, respectively. These bands are commonly reported for silane-modified CNCs and are consistent with the silanized material supplied by the manufacturer [26,53] (main manuscript).

 https://doi.org/10.3390/polym18131665

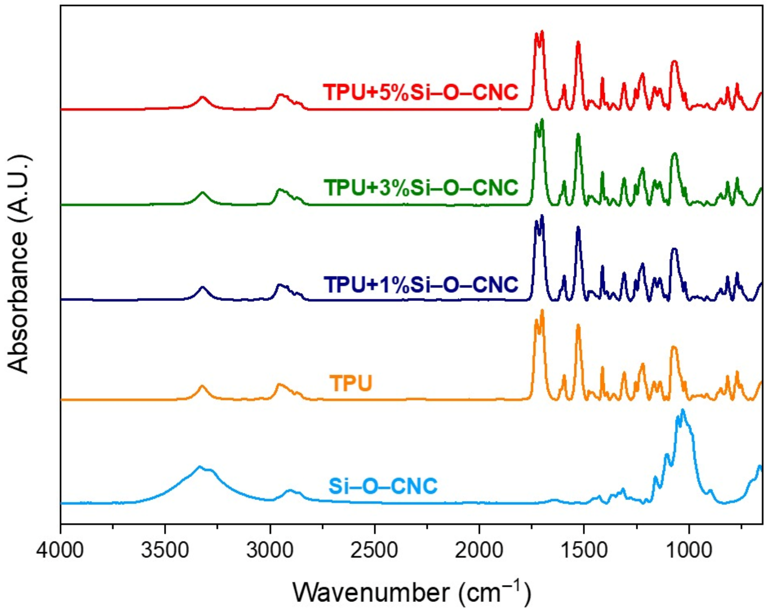

Figure S1. FTIR spectra of pristine TPU, Si–O–CNC and TPU/Si–O–CNC nanocomposites.

Table S1. Vibrational assignment of the FTIR spectra for pristine TPU and TPU/Si–O–CNC nanocomposites.

**Assignment**	**Groups Assignment**	**Sample**			
		**TPU**	**TPU +1% Si–O–CNC**	**TPU +3% Si–O–CNC**	**TPU +5% Si–O–CNC**
υ_{st} **N–H**	*free* NH-groups	3325	3323	3324	3324
υ_{st} **C–H₂**	CH_2 groups	2956-2875	2956-2873	2955-2872	2955-2872
υ_{st} **C=O**	*free*-C=O	1728	1727	1727	1727
υ_{st} **C=O**	*H*-bonded C=O	1701	1702	1702	1702
υ_{st} **C=O**	C=O Urea	-	1637	1638	1639
υ_{st} **C=C**	Aromatic ring vibration	1596	1596	1596	1596
δ **N–H**	NH bending	1529	1529	1529	1529
δ **C–H**	CH_2 bending	1478-1361	1478-1362	1478-1362	1478-1362
υ_{st} **Amide III**	C-N + C=O combined stretching	1254	1254	1254	1254
δ **C–N**	C-N bonded	1309-1219	1309-1220	1309-1220	1309-1220
δ **CO–O–C**	CO-O-C bending	1075	1075	1075	1075
δ **C–O–C**	C-O-C bending	770	770	770	770

Higher-magnification SEM images acquired at 2000x are displayed in **Figure S2** to better assess the of absence of visible micron-scale agglomerates.

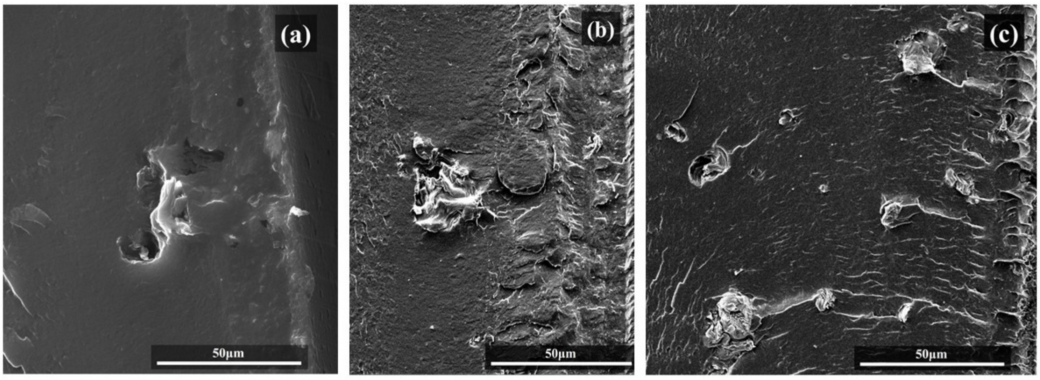

Figure S2. Scanning Electron Microscopy (SEM) images of (**a**) TPU+1%Si–O–CNC, (**b**) TPU+3%Si–O–CNC, (**c**) TPU+5%Si–O–CNC at higher magnification.

https://doi.org/10.3390/polym18131665
